# Strain and Pressure Sensors Based on MWCNT/PDMS for Human Motion/Perception Detection

**DOI:** 10.3390/polym15061386

**Published:** 2023-03-10

**Authors:** Xin Zhao, Dong Mei, Gangqiang Tang, Chun Zhao, Jianfeng Wang, Minzhou Luo, Lijie Li, Yanjie Wang

**Affiliations:** 1 Jiangsu Provincial Key Laboratory of Special Robot Technology, Changzhou Campus, Hohai University, Changzhou 213022, China; 2 Multidisciplinary Nanotechnology Centre, College of Engineering, Swansea University, Swansea SA1 8EN, UK

**Keywords:** MWCNT/PDMS, strain sensor, pressure sensor, wearable devices, smart glove

## Abstract

Flexible wearable devices have attracted wide attention in capacious fields because of their real-time and continuous monitoring of human information. The development of flexible sensors and corresponding integration with wearable devices is of great significance to build smart wearable devices. In this work, multi-walled carbon nanotube/polydimethylsiloxane-based (MWCNT/PDMS) resistive strain sensors and pressure sensors were developed to integrate a smart glove for human motion/perception detection. Firstly, MWCNT/PDMS conductive layers with excellent electrical and mechanical properties (resistivity of 2.897 KΩ · cm, elongation at break of 145%) were fabricated via a facile scraping-coating method. Then, a resistive strain sensor with a stable homogeneous structure was developed due to the similar physicochemical properties of the PDMS encapsulation layer and MWCNT/PDMS sensing layer. The resistance changes of the prepared strain sensor exhibited a great linear relationship with the strain. Moreover, it could output obvious repeatable dynamic response signals. It still had good cyclic stability and durability after 180° bending/restoring cycles and 40% stretching/releasing cycles. Secondly, MWCNT/PDMS layers with bioinspired spinous microstructures were formed by a simple sandpaper retransfer process and then assembled face-to-face into a resistive pressure sensor. The pressure sensor presented a linear relationship of relative resistance change and pressure in the range of 0–31.83 KPa with a sensitivity of 0.026 KPa^−1^, and a sensitivity of 2.769 × 10^−4^ KPa^−1^ over 32 KPa. Furthermore, it responded quickly and kept good cycle stability at 25.78 KPa dynamic loop over 2000 s. Finally, as parts of a wearable device, resistive strain sensors and a pressure sensor were then integrated into different areas of the glove. The cost-effective, multi-functional smart glove can recognize finger bending, gestures, and external mechanical stimuli, which holds great potential in the fields of medical healthcare, human-computer cooperation, and so on.

## 1. Introduction

Flexible smart wearable devices are the crystallization of intelligent design for daily wearables, which come in various forms to adapt to different parts of the human body [1]. As the core component of smart wearable devices, the form and function of sensors directly affect the effective and comfortable application of smart wearable devices. With the rapid development of information technology and material science, smart wearable devices are required to have as high flexibility, adaptability, and excellent sensing performance as possible. Therefore, flexible sensors [2,3] have gradually replaced traditional sensors based on hard substrates. At present, flexible sensors can be divided into several typical types according to their working principles, such as piezoelectric [4], piezoresistive [5], capacitive [6], ionic [7], triboelectric [8], etc. Among them, flexible piezoresistive sensors can be manufactured in various modes through different combinations of elastic substrate materials and micro-nano conductive media, which have the advantages of a simple structure, easy manufacturing process, low cost, high sensitivity and a wide response range. The resistive sensors with flexible substrates such as silicone, PDMS, and gel are closer to the mechanical properties of human skin by virtue of their flexibility and high ductility [9,10,11].

At present, resistive sensors are mostly prepared by depositing a conductive layer that can produce resistance changes on the surface of flexible substrate films such as PDMS or silica gel [12,13,14]. Under the external stimuli, the conductive particles in the conductive layer are deformed to be broken or overlapped with each other, finally showing the change of readable resistance signals. Amjadi et al. [15] developed a strain sensor based on an Ag nanowires (AgNWS) elastomer nanocomposite by embedding AgNWS film between two PDMS layers that have a stretching rate of 70%. Yoon et al. [16] sprayed carbon nanotubes onto the surface of silica gel as a conductive substance to form a piezoresistive strain sensor that can fit human skin and achieve about 500% tensile properties. Zhou et al. [17] embedded single-walled carbon nanotube fragments (SWCNT) in polydimethylsiloxane (PDMS) to develop a scalable strain sensor that maintains its sensitivity at high strain levels. The means of conductive coating together with flexible substrates can enhance the large deformation response of resistive sensors. However, the obvious hierarchical division between the electrode layer and the substrate layer and the low structural integration easily produce the electrode shedding phenomenon under external contact or internal extrusion, which brings a negative influence on the sensing performance and signal post processing [18]. In general, the resistive strain sensor has a weak pressure-sensing ability due to the compactness in the internal structure and thickness limitation. Researchers have found that the pressure response characteristics of the resistive sensor can be enhanced by introducing the surface microstructure and the internal porous structure [19]. Appropriate surface structure treatment on the flexible substrate can effectively improve the sensing performance of resistive pressure sensors, including micro-pillar [20], pyramid structure [21], microsphere structure [22], plant-like surface microstructure [23], and interlocking nanopillar array structure imitating human hair [24]. Ma et al. [25] fabricated a piezoresistive sensor based on ultra-light and super-elastic MX/rGO aerogel using the hybrid, three-dimensional structure of MXene and reduced graphene oxide and their pressure-sensitive characteristics, which can easily capture external stimulus signals below 10 Pa. Xu et al. [26] proposed an all-solution-processed multi-mode biomimetic stretchable e-skin with a layered face-to-face asymmetric microstructure consisting of a PEDOT: PSS protective layer, an AgNWS conductive layer, and a silicone rubber matrix, which can achieve a variety of stimulus sensing including pressure and strain. Pang et al. [27] proposed that the bioinspired random distribution spinosum (RDS) microstructure could effectively enhance the sensitivity and detection limit of the resistive pressure sensor and then developed a pressure sensor based on graphene coating with a sensitivity of 25.1 KPa^−1^ and linear range of 0–2.6 KPa. Although conductive coatings such as AgNWS and CNT can be transferred to the surface of the flexible substrate by sputtering or in-situ polymerization, the conductive particles are easy to agglomerate in the local region of the microstructure due to the low elastic modulus of the substrate and the existence of microstructure, thus affecting the complete transfer of thin and dense conductive coatings. In addition, it is easy to form ohmic contact with high resistance at the contact surface between the conductive layer and the substrate using spraying method, resulting in obvious additional impedance that affects the performance of the sensors and limits their applications in wearable devices.

Herein, MWCNT/PDMS conductive layers with excellent electromechanical properties were fabricated based on multi-walled carbon nanotubes and polydimethylsiloxane, and the integrated fabrication process of resistive strain sensors was developed. The homogeneous nature of the PDMS encapsulation layer and the MWCNT/PDMS sensing layer endows the sensor with overall structural stability. The resistance strain sensor can generate obvious and continuous dynamic responses under bending/restoring and stretching/releasing behaviors, and the relative resistance change has a significant linear relationship with the strain in the range of 0 to 100%. In addition, a resistive pressure sensor with high sensitivity and wide linear range was fabricated by introducing a biomimetic spinous microstructure using a simple and fast microstructure transfer method. As a part of wearable smart gloves, the resistive strain sensors were woven into the knuckles of the fabric gloves to decode and transmit gesture language, and a resistive pressure sensor was integrated into the back of the hand to sense pressure signals. The highly integrated smart glove can be used for real-time monitoring of joint movement and placement signals, and visual signals can be read on a PC device, which has broad application prospects in the fields of motion monitoring, medical healthcare, and human–computer interaction.

## 2. Materials and Methods

### 2.1. Materials

The multi-wall carbon nanotube (MWCNT) (diameter of 10–20 nm, length of 10–30 μm, purity ≥ 95%) was purchased from Nanjing XFNANO Materials Tech Co., Ltd. (Nanjing, China). Sylgard 184 polydimethylsiloxane (PDMS) including a silicone-elastomer base liquid and curing agent was purchased from Dow Corning Chemical Company (Midland, MI, USA). Dimethylsilicone fluid was purchased from Dow Corning Chemical Company (Midland, MI, USA). Silver conductive adhesive was purchased from the KITSON Metal Products Co., Ltd. (Guangzhou, China). The VHB^TM^ tape (thickness 0.13 mm) and self-adhesive elastic bandage were purchased from Minnesota Mining and Manufacturing Corporation company (Shanghai, China). Glove, conductive tapes and needlework were purchased from local stores (Changzhou, China). It is worth noting that the conductive tapes are made of high-strength polyester fiber cloth plated with high-conductivity copper-nickel metal, which have excellent conductivity and adhesion. Compared with copper wires, the conductive tapes as extraction electrode deform do not interfere with the signal.

### 2.2. Fabrication

**Figure 1:**
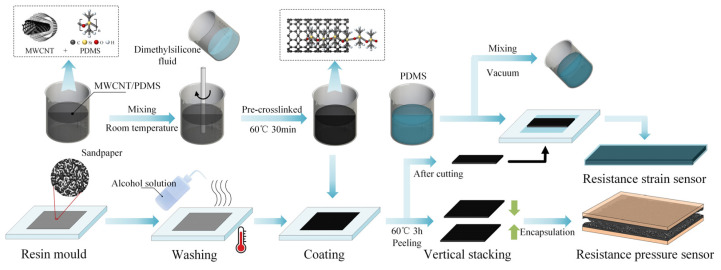
Schematic diagram of resistance strain and pressure sensors preparation process.

The silicone-elastomer base liquid and curing agent were mixed in a weight ratio of 10:1 to obtain the PDMS solution. Afterward, the MWCNT, in amounts of 0.2023 g, was fully mixed with 2 g of PDMS solution and 0.1264 g of dimethylsilicone fluid. Dimethylsilicone fluid as the dispersant, with the same physicochemical properties as PDMS liquid, can enhance the dispersity of MWCNT in PDMS and improve electrical conductivity and mechanical properties of the MWCNT/PDMS layer [28]. After being pre-crosslinked at 60 °C for 30 min, MWCNT (8 wt%) and PDMS polymer crosslinked each other to form a stable paste mixture. The final mixture was poured into a resin mold with a dimension of 35 mm length × 35 mm width × 1 mm height and heated in a vacuum drying oven at 60 °C for 3 h. After the curing process, the MWCNT/PDMS layer was carefully peeled off from the resin mold, then the MWCNT/PDMS layer was cut into small sheets of 30 mm length, 10 mm width, and 1 mm thickness. Conductive tapes were connected to the surface of the MWCNT/PDMS layer at both ends using silver conductive adhesive. After that, the PDMS solution was prepared repeatedly, and bubbles in the mixture were extracted by a vacuum pump. The above PDMS solution was coated to cover the MWCNT/PDMS layer in the resin mold to obtain a resistance strain sensor with a stable homogeneous structure that can be attributed to the homogeneity structure of the PDMS encapsulation layer and MWCNT/PDMS sensing layer [29]. As for the resistance pressure sensor, the sandpaper (roughness of 800 mesh, thickness of 0.2 mm) was used as a template to transfer the bioinspired spinous microstructure. Subsequently, MWCNT/PDMS mixture was coated on pretreated sandpaper with alcohol solution to obtain the randomly distributed spinous microstructure, which was then stripped and stacked face-to-face to form a spinous interlock structure. Note that only one side of the PDMS/MWCNT layer had the microstructure, whereas the other side was plain. The sandpaper and mold were tightly pasted with VHB to prevent curling during heating. The detailed preparation process of the strain and pressure sensors-based MWCNT/PDMS layer is shown in Figure 1.

### 2.3. Characterization and Measurements

**Figure 2:**
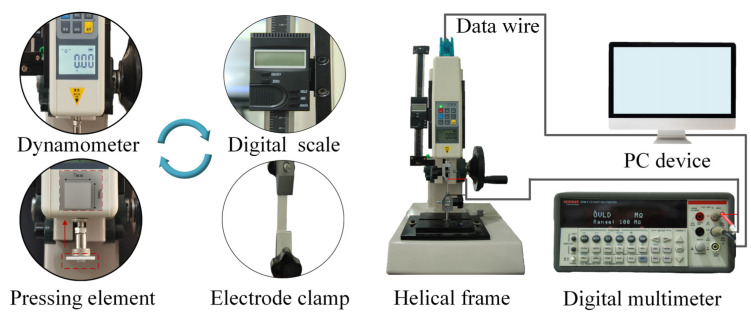
Schematic diagram of a test platform for resistive strain and pressure sensor.

A self-built sensing test platform was used to characterize the sensing performance of resistance sensors, which consists of a push-pull dynamometer (HP-500, Edelberg Instruments Co., Ltd., Beijing, China.), a digital multimeter, and a personal computer, as shown in Figure 2. According to different working modes of the resistance sensor, a stretching element and a pressing element were respectively arranged on the dynamometer, and the resistance changes under tensile deformation or pressure load were recorded by a digital multimeter (2100 6 1/2, KEITHLEY Company, Solon, OH, USA) and then transmitted to a PC device. Two stretching elements (electrode clamps) were respectively fixed on the dynamometer to provide constraints for both ends of the resistive strain sensor. The amount of strain is calculated by $\varepsilon =\Delta L⁄L_{0}\;$, and ΔL was recorded by digital scale. As for the resistive pressure sensor, the upper surface of which was parallel to the pressing element, the pressure with different amplitudes can be regulated by operating the helical frame, and the numerical value of the load was read through a digital display screen of the dynamometer. The mechanical performance of the prepared MWCNT/PDMS layers was tested at room temperature on an electronic universal experimental machine with loading speeds at 10 mm/min. The dynamic response of the resistive strain sensor to the bending/restoring and stretching/releasing behaviors were realized by controlling the loading displacement of a stepper motor. The dynamic load applied to the resistive pressure sensor artificial receptor was generated by a vibration exciter (SA-JZ002). The surface microstructure of the fabricated MWCNT/PDMS layers was characterized by an electron microscope.

### 2.4. Integration of the Sensors on a Glove

**Figure 3:**
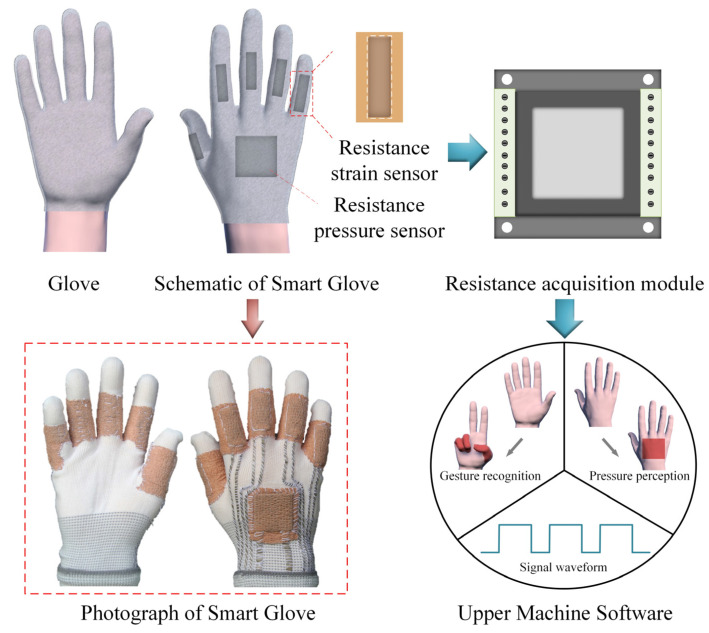
Integration of the resistive sensors with a smart glove for gesture recognition and perception detection.

A glove, sensor modules, signal processing system, and upper machine software together constitute a wearable smart glove system. Five resistive strain sensors were successfully fixed on the knuckles of a fabric glove by sewing method combined with medical elastic bandage to provide constraints. To achieve a stable electrical connection, conductive tapes were attached to the fabric glove by sewing. The packaged resistive pressure sensor was fixed on the back of the glove in the same way. The signal processing system was mainly composed of a power supply module and a multi-channel resistance acquisition module. Both conductive tapes were connected to a resistance acquisition module for signal transmission and analysis. Finally, the real-time resistance change of the smart glove signal was obtained at the upper machine software. The schematic diagram of the smart glove system is shown in Figure 3.

## 3. Results and Discussion

### 3.1. Performance Testing of MWCNT/PDMS Layer

**Figure 4:**
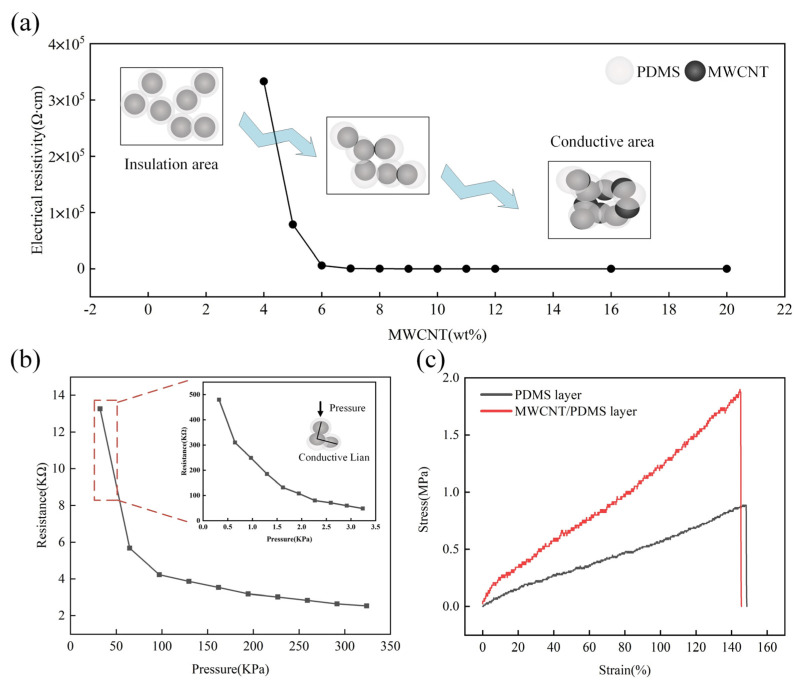
(**a**) Percolation curve of MWCNT/PDMS conductive layer; (**b**) resistance variation of MWCNT/PDMS layer under high-pressure range (0–330 KPa) and under low–pressure range (0–3.3 KPa); and (**c**) stress-strain curves of pure PDMS layer and MWCNT/PDMS layer.

Percolation characteristics are ubiquitous in particle-filled polymer composites [30], which are related to the properties of conductive fillers, such as aspect ratio, conductivity, dispersion, etc. To evaluate the conductivity of the MWCNT/PDMS layer, a series of samples were prepared with a gradient increase of the MWCNT content from 2 wt% to 20 wt%; the percolation curve is shown in Figure 4a. There were three samples with the same MWCNT content for the experiment, then the average value was taken. It can be seen that the MWCNT/PDMS layer begins to be conductive when the content of the MWCNT reaches 4 wt%. The value of the content of the conductive filler, which transforms the polymer from an insulating object to a conductive object, is called the percolation threshold. After that, with the increase of MWCNT content, the conductive network inside the polymer becomes more compact, so the resistivity decreases gradually while the conductivity improves. In this work, the MWCNT/PDMS layer with 8 wt% filling content was utilized for the preparation of the sensor. Two MWCNT/PDMS layers with spinous microstructures were assembled face-to-face and placed on the sensing test platform. The resistance changes of the assembled MWCNT/PDMS device under a progressive pressure load of 0–330 KPa are shown in Figure 4b. The resistance of the assembled MWCNT/PDMS device decreases with the increase of the applied load, showing a good piezoresistive property, which can be attributed to the increase of the contact area among the microstructures under external load, resulting in the increase of conductive paths. Similarly, the piezoresistive behavior of the assembled MWCNT/PDMS device was tested under a load of 0–3.3 KPa, as shown in the enlarged view of Figure 4b. The MWCNT/PDMS layer with 8 wt% MWCNT content exhibits more linear resistance changes and higher sensitivity under small loads. There were three samples for each experiment, then the average value was taken. In addition, the appropriate amount of MWCNT can effectively enhance the mechanical properties of conductive films [31]. The stress-strain curves of the pure PDMS layer and the MWCNT/PDMS layer are shown in Figure 4c. The elastic modulus and the breaking elongation of the pure PDMS layer are 5.51 KPa and 148%, respectively, whereas those of the MWCNT/PDMS layer is 11.59 KPa (increased by 110%) and 145% (decreased by only 2.02%). This can be attributed to the fact that the large aspect ratio of MWCNT enhances the crosslink density between polymer chains, so the elastic modulus increases and the elongation at break decreases slightly. The above results demonstrate that the MWCNT/PDMS layer with an MWCNT content of 8 wt% has excellent conductive and mechanical properties.

**Figure 5:**
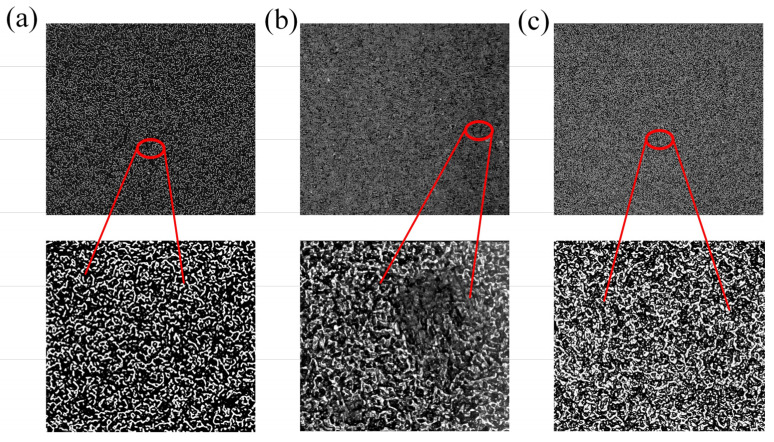
(**a**) Microstructure of 800 mesh sandpaper; (**b**) surface microstructure of MWCNT/PDMS layer without oily release agent in fabrication; and (**c**) MWCNT/PDMS layer with complete spinous microstructure.

Due to the large aspect ratio of MWCNT fillers, it is easy to increase the viscosity of the conductive film and strengthen the adhesion between the film and the substrate, resulting in local structural defects in the demoulding process and weakening the mechanical properties of the MWCNT/PDMS layer, as shown in Figure 5b. Therefore, in the process of preparing the MWCNT/PDMS layer, the oily release agent was used on the surface of the sandpaper substrate to protect the surface microstructure of the MWCNT/PDMS mixture film from failure. It can be seen from Figure 5c that the presence of the release agent improves the lubricity between the conductive film and the sandpaper substrate. The surface microstructure of the sandpaper (Figure 5a) was transferred to the surface of the MWCNT/PDMS layer completely, densely, and uniformly, which provides a guarantee for the further development of resistive pressure sensors.

### 3.2. Sensing Properties

**Figure 6:**
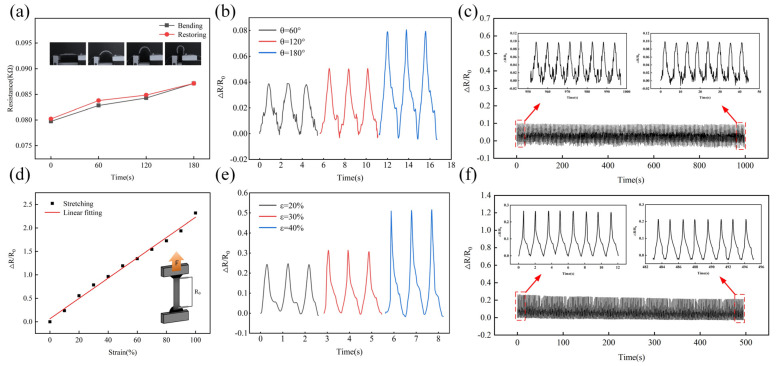
(**a**) Relative resistance change of strain sensor during bending/restoring process; (**b**) dynamic response of strain sensor under different angle bending/restoring cycles; (**c**) cyclic stability of strain sensor under 180° bending/restoring cycles; (**d**) relative resistance change of the strain sensor during stretching process; (**e**) dynamic response of strain sensor under different strain stretching/releasing cycles; and (**f**) cyclic stability of strain sensor under 40% stretching/releasing cycles.

To verify the sensing characteristics of the resistive strain sensor, the strain response characteristics of the sensor under bending and stretching were tested. For each experiment, the same sample was tested three times, then the average value was taken. The strain sensor was fixed on the spring steel connected with a stepper motor. With the movement of the motor, the sensor generates bending/restoring cycle behavior, which realizes the curvature characterization of the strain sensor. Figure 6a shows the relative resistance change of the resistive strain sensor fixed on the bending test platform in the bending states of 0°, 60°, 120°, and 180°. The bending angles were obtained by a digital display angle measuring tape. When the bending angle increases from 0° to 180°, the resistance of the strain sensor increases linearly from 0.079 KΩ to 0.087 KΩ due to the change of the internal conductive network caused by strain, whereas the resistance decreases during the recovery process. Note that the resistance of the strain sensor in the restoring process is greater than that in the bending process, such as the increase of nearly 1.147% at 60°, which is attributed to the response hysteresis phenomenon. However, the resistance change is within the allowable error range, indicating that the strain sensor has good reversible characteristics. Figure 6b shows the dynamic response signals of the strain sensor at different bending angles from 60° to 180°. The amplitude of the resistance response gradually increases, indicating that there is a good dependence between the response signal and the bending angle. The amplitude and the peak shape of the response signal are consistent under three bending/restoring behaviors at the same angle, thereby showing excellent signal repeatability. Figure 6c shows the signal response of the strain sensor after 180° bending/restoring cycling behavior with a duration of 1000 s. The relative resistance difference $\Delta R/R_{0}$ remains at ~0.1 with no significant change (with a repeatability error of 0.6%). As can be seen from the magnified illustration, even after a bending/restoring cycle of 1000 s cycle period, the $\Delta R/R_{0}$ value of the strain sensor is almost the same as the first round, which confirms the long-term structural stability and sensing reliability in the bending/recovery state. In the tensile test, the dependence of resistance change ratio $\Delta R/R_{0}$ and tensile strain $\varepsilon =\Delta L/L_{0}$ is monitored to test the performance of the strain sensor. Figure 6d shows the relative resistance change of the strain sensor upon increasing strain, when the sensor is stretched to a strain ε, the increase ΔL leads to a reduction in the conductive path inside the sensor and a lengthening of the electron transfer path, thus increasing the total resistance under tensile load. When the applied strain increases from 0% to 100%, the relative resistance ($\Delta R/R_{0}$) varies linearly with a slope of 0.022, indicating that the sensor has good linear sensing performance with a sensitivity of 0.022 KPa^−1^. Figure S1 shows the hysteresis loss of the resistive strain sensor during the stretching/releasing process. It can be seen from Figure 6e that the strain sensor can ensure a stable and distinguishable signal response at different tensile strains of 20%, 30%, and 40%, which has excellent repeatability. To investigate the cyclic durability and repeatability, the strain sensor was subjected to stretching/releasing cycles at 40% strain for 500 s, as shown in Figure 6f. Obviously, the strain sensor can stably output a resistance response signal after a long-term dynamic stretching/releasing cycle, and the $\Delta R/R_{0}$ of the adjacent eight response signals keep good repeatability and good response stability (with a repeatability error of 10.90%).

**Figure 7:**
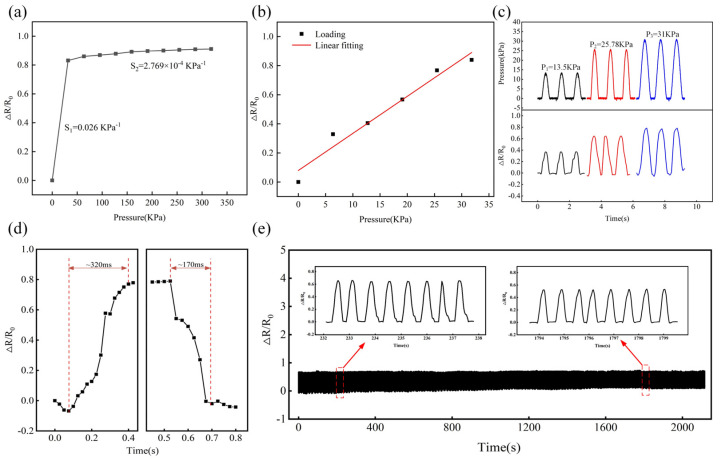
(**a**) Relative resistance change of pressure sensor during loading process; (**b**) high-resolution curve for low-pressure range 0–31.83 KPa; (**c**) dynamic response of pressure sensor under loading/unloading cycle with different pressure; (**d**) response and recovery time of pressure sensor at loading/unloading pressure of 31 KPa; and (**e**) cyclic stability of pressure sensor under 40% loading/unloading cycles.

To study the sensing characteristics of the resistive pressure sensor, normal loads with different magnitudes were applied to the sensor surface. The same sample was tested three times for each experiment, and from the data were taken the average. Figure 7a shows the variation curve of the relative resistance of the resistance pressure sensor with the normal pressure. It can be seen that the variation of the relative resistance presents two great linear response regions with the increase of the loading pressure. With the increase of the loading amplitude from 0 KPa to 31.83 KPa, the resistance of the pressure sensor decreases sharply from 4.5 KΩ to 0.76 KΩ, showing a high sensitivity (S_1_ = 0.026 KPa^−1^). The resistance of the pressure sensor decreases slowly after the load amplitude is over 31.83 KPa, and the sensitivity is S_2_ = 2. 769 × 10^−4^ KPa^−1^. Figure 7b shows a high-resolution scatter plot of the relative resistance of the pressure sensor as a function of pressure in the low-pressure range, and the sensitivity obtained through linear fitting is consistent with the calculated sensitivity S_1_. The high sensitivity in the low-pressure range (0 KPa to 31.83 KPa) is attributed to the large change in the effective contact area between the spinous microstructure of two MWCNT/PDMS layers, then the conductive path increases rapidly, thus resulting in a large resistance variation. With the increase in pressure, the contact between the microstructures on the sensor gradually reaches saturation, whereas the change of intrinsic resistance is inconspicuous due to the limitation of material thickness, so the sensitivity of the pressure sensor decreases sharply in the high-pressure range (>31.83 KPa). A circular pressing element of R = 10 mm was used to carry out cyclic loading/unloading tests on the pressure sensor under pressure with different amplitudes. The dynamic response signals are shown in Figure 7c. With the dynamic load amplitude increase, the pressure sensor shows more obvious dynamic response signals. Moreover, the waveform of the response signal is consistent with the pressure signal. It indicates that the pressure sensor has great dynamic response characteristics under different pressure. Furthermore, response time and cycle stability also play important roles in the application of pressure sensors. The normal pressure is applied by a vibration exciter with a frequency of 1 Hz and an amplitude of 31 KPa. As shown in Figure 7d, the loading response time of the pressure sensor is 320 ms and the release response time is 170 ms. The resistance signal changes faster during the pressure unloading process. To further test the cyclic stability of the pressure sensor, a pressure of 25.78 KPa was repeatedly applied to the sensor for a duration of about 2000 s. The fluctuation of the relative resistance change was very small, as shown in Figure 7e. At the same time, the enlarged illustration clearly shows that the response signals of adjacent periods of the pressure sensor have almost the same amplitude and peak shape, confirming the high repeatability and good cycle stability of the pressure sensor (with a repeatability error of 17.70%).

### 3.3. A Smart Glove for Human Motion/Perception Detection

**Figure 8:**
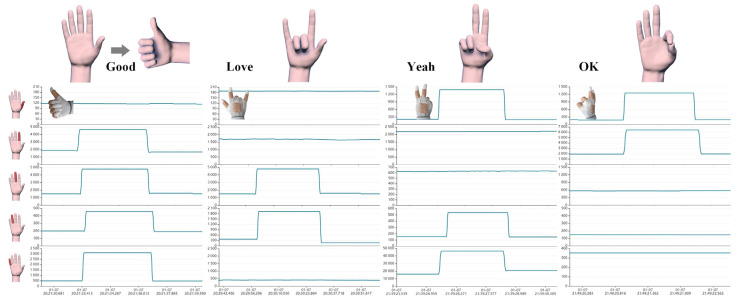
Demonstration of practical application: gesture recognition.

The real-time detection of joint motion and pressure perception employing an integrated wearable device is of great value for assisting in diagnosis and monitoring rehabilitation status. Due to the great sensing properties and mechanical performances of the resistive strain sensor based on MWCNT/PDMS, the smart glove can be used for gesture recognition. When completing the different gestures, each finger has diverse bending states and the corresponding resistance change, which can be easily recognized by analyzing the bending signals of five fingers. Figure 8 shows the resistance change curves of the smart glove in four gestures. For example, in the “Love” gesture, the middle and ring fingers are bent, whereas the other fingers are unbent. The resistance change of each finger on the smart glove is plotted as the time function of the corresponding gesture, demonstrating the ability to track the movement.

**Figure 9:**
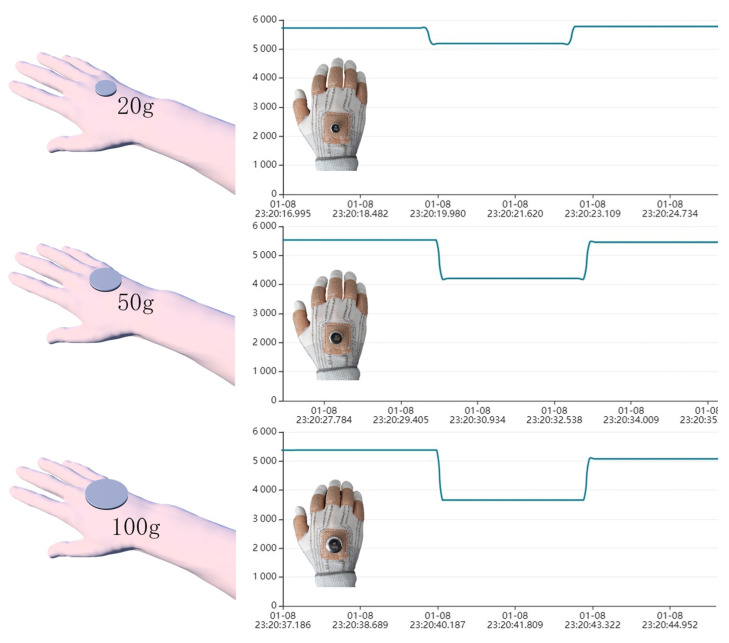
Demonstration of practical application: pressure signal monitoring.

Furthermore, the performance of the smart glove in perception detection was examined using the same signal processing system. The back of the human hand also has the function of perception, but the existing smart wearable devices do not involve related applications. Herein, the smart glove was equipped with a resistive pressure sensor on the back of the hand that can monitor the placement signals of different weights, as shown in Figure 9. When a round object with a weight of 20 g was placed on the back of the smart glove, the response resistance decreased significantly from the initial state until it returned to the initial state after the weight was removed. Furthermore, the amplitude of the response resistance signal also increases along with the gradually increasing weight of the object, thereby demonstrating the capability of monitoring the pressure signal on the back of a hand.

## 4. Conclusions

In this work, MWCNT/PDMS layers with excellent electromechanical properties were obtained by means of a facile and low-cost scraping-coating method that can achieve large-area rapid fabrication. The elastic modulus of the MWCNT/PDMS layer was enhanced by 110% compared with that of the pure PDMS layer. The resistive strain sensor developed based on the homogeneity of the PDMS encapsulation layer and MWCNT/PDMS sensing layer has a stable integrated structure that exhibited a great linear relationship between resistance changes and strain. The strain sensor still exhibited excellent repeatability, stability, and durability after 180° bending/restoring cycling behavior with a duration of 1000 s. Under stretching/releasing cycling behavior, a linear relationship between the resistance changes and the strain is established with a sensitivity of 0.022 KPa^−1^ in the strain range from 0% to 100%. After the 40% stretching/releasing cycling behavior lasted for 500 s, the strain sensor could still stably output the resistance response signal. In addition, inspired by the interlocking microstructure of the epidermis and dermis boundaries in human skin, a MWCNT/PDMS-based resistive pressure sensor with spinous microstructure was successfully fabricated by a simple sandpaper stencil printing process. The sensitivity of the pressure sensor is as high as 0.026 kPa^−1^ with a linearity range of up to 31.83 KPa. Then, the resistance changes with the sensitivity of 2.769 × 10^−4^ KPa^−1^ continues to increase linearly with the accretive load. The pressure sensor responded quickly and showed good stability after 25.78 KPa loading/unloading cycling behavior beyond the 2000 s. Finally, a smart glove was developed via integrating the resistive strain sensor and the resistive pressure sensor with a fabric glove, cooperating with the signal processing system. The smart glove could accurately identify the motion of finger joints, which provides a monitoring function for the rehabilitation of patients with hand disorders. The pressure-sensing signal on the back of the hand realizes the further diversified perception of the smart glove. The potential of resistive sensors based on MWCNT/PDMS as the core sensing module of wearable devices was proven by excellent applications in a smart glove for human motion/perception detection.

## Data Availability

Not applicable.

## Supplementary Materials

The following supporting information can be downloaded at: https://www.mdpi.com/article/10.3390/polym15061386/s1, Figure S1: The hysteresis loss of resistive sensor during stretching/releasing process.
