# Supplementary material for: Strain and Pressure Sensors Based on MWCNT/PDMS for Human Motion/Perception Detection
Source: Polymers (Basel). 2023 Mar 10;15(6):1386. doi: 10.3390/polym15061386 (PMC10055516; doi:10.3390/polym15061386)
Supplement: Supplementary file 1 [file polymers-15-01386-s001.zip › polymers-2220211-supplementary.pdf]

Supplementary Materials

Strain and Pressure Sensors Based on MWCNT/PDMS for Human Motion/Perception Detection

For each experiment, the same sample was tested three times, then the average value is taken. Figure S1 shows the resistance change of the resistive strain sensor during stretching/releasing process from 0% to 100%. During the releasing process, due to the elastic hysteresis loss of the material, it fails to return to the deformation state during the stretching process and shows a larger resistance response. The maximum hysteresis loss of the strain sensor is 10.65% in the hysteresis curve within the strain range of 0%-100%, and the resistance change in the stretching/releasing process is close to linear, which shows that the sensor has good reversible characteristics.

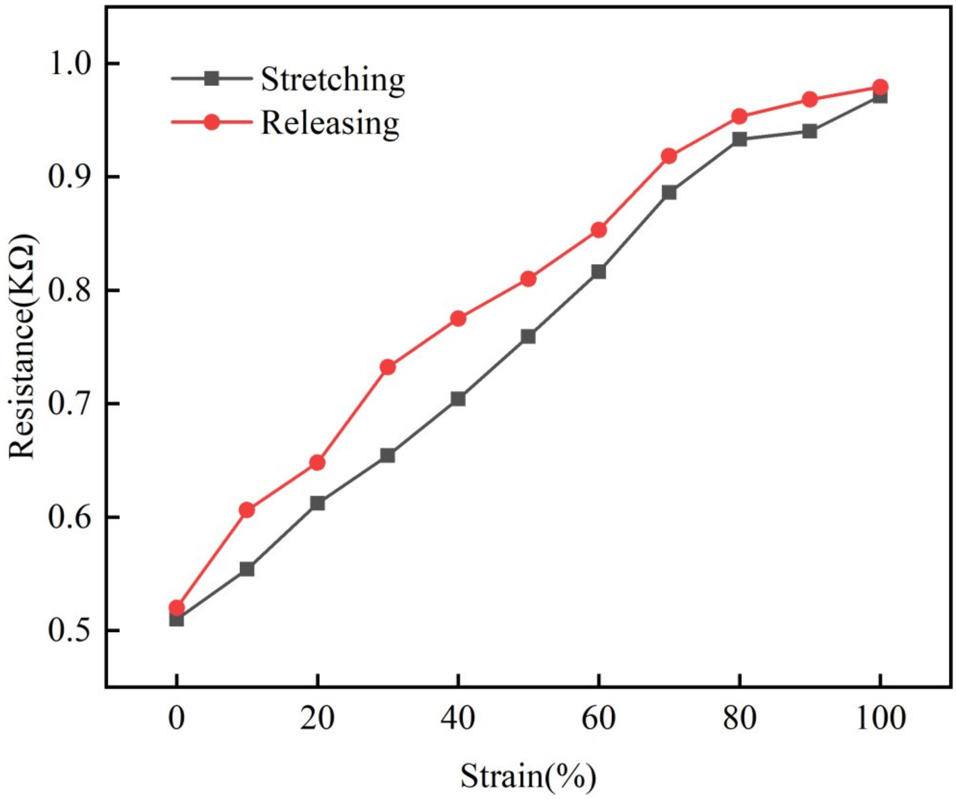

Figure S1. The hysteresis loss of resistive sensor during stretching/releasing process.
